# Mechanisms of Alpha-Synuclein Action on Neurotransmission: Cell-Autonomous and Non-Cell Autonomous Role

**DOI:** 10.3390/biom5020865

**Published:** 2015-05-13

**Authors:** Marco Emanuele, Evelina Chieregatti

**Affiliations:** Department of Neuroscience and Brain Technologies, Istituto Italiano di Tecnologia, 16163 Genoa, Italy; E-Mail: marco.emanuele@iit.it

**Keywords:** alpha-synuclein, exo/endocytosis, calcium entry, non-cell autonomous, lipid microdomains

## Abstract

Mutations and duplication/triplication of the alpha-synuclein (αSyn)-coding gene have been found to cause familial Parkinson’s disease (PD), while genetic polymorphisms in the region controlling the expression level and stability of αSyn have been identified as risk factors for idiopathic PD, pointing to the importance of wild-type (wt) αSyn dosage in the disease. Evidence that αSyn is present in the cerebrospinal fluid and interstitial brain tissue and that healthy neuronal grafts transplanted into PD patients often degenerate suggests that extracellularly-released αSyn plays a role in triggering the neurodegenerative process. αSyn’s role in neurotransmission has been shown in various cell culture models in which the protein was upregulated or deleted and in knock out and transgenic animal, with different results on αSyn’s effect on synaptic vesicle pool size and mobilization, αSyn being proposed as a negative or positive regulator of neurotransmitter release. In this review, we discuss the effect of αSyn on pre- and post-synaptic compartments in terms of synaptic vesicle trafficking, calcium entry and channel activity, and we focus on the process of exocytosis and internalization of αSyn and on the spreading of αSyn-driven effects due to the presence of the protein in the extracellular milieu.

## 1. Introduction

**Figure 1:**
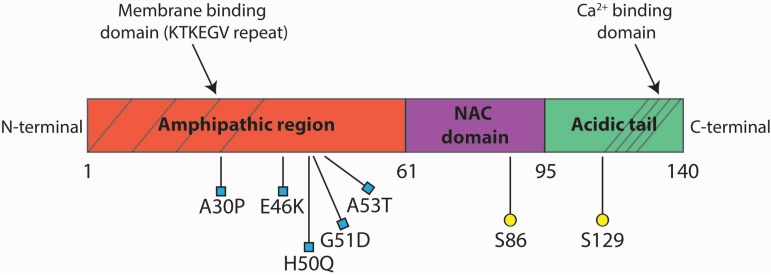
Structure of the αSyn protein. The N-terminal domain (red) is composed of KTKEGV repeats where human missense mutations associated to familial PD have been found. The central hydrophobic core (purple) is called NAC domain and promote aggregation of the protein. The C-terminal domain (green) is the acidic tail that contains phosphorylation sites and the calcium binding site.

Alpha-synuclein (αSyn), a small protein of 140 amino acids specifically enriched in the presynaptic nerve terminals [1], has been found as a major component of Lewy bodies, with intraneuronal inclusion present in the brain of Parkinson’s disease (PD) patients [2,3]. Human αSyn protein consists of three distinct structural motifs. The scheme in Figure 1 depicts αSyn’s structure. The N-terminal region (residues 1–61) contains four of the seven imperfect repeats of the KTKEGV motif, reminiscent of the lipid-binding domain of apolipoproteins, which, in certain conditions, forms amphipathic helices that associate with vesicles containing phospholipids [4], such as the synaptic vesicles membrane, both *in vitro* and *in vivo*. It is the region where missense mutations linked to early onset familial PD have been found. Binding to the membrane determines a stabilization of the protein structure, due to the increase in amphipathic α-helix content from 3% to over 70%. Mutations in the sequence coding for the N-terminal domain determined a mislocalization of αSyn and a detachment from the plasma membrane in yeast [5]. The central core (residues 62–95) is a hydrophobic region (non-amyloid-β component or NAC domain), which is responsible for aggregation and toxicity. The NAC domain comprises the highly amyloidogenic part of αSyn [6,7], which mediates its conformational change from a random coil to a β-sheet structure. The C-terminus (residue 96–140), rich in acidic and proline residues, interacts with several proteins [8] and contains sixteen residues repeats, which could be important for Ca^2+^-binding [9]. The C-terminal region tends to decrease protein aggregation, displaying an opposite effect to the NAC domain. Indeed, truncated forms of αSyn lacking the C-terminal tail (αSyn 1–120) are more prone to aggregation [10,11]. Many posttranslational modifications of αSyn occur at the C-terminus, such as phosphorylation of Ser129 or nitration of Tyr125, Tyr133 and Tyr136. These modifications may alter αSyn protein conformation, promoting oligomerization and filament formation [12,13]. αSyn belongs to the intrinsically-disordered protein (IDP) family, a group of proteins that lack an organized secondary structure [14]. For its peculiar propensity to undergo structural changes, according to the different environment in which it is found, Syn has been referred to as a “protein chameleon” [15]. For this reason, Syn may be unfolded in solution, may assume an α-helical structure when bound to lipids or may fold into β-sheet-like structures, when aggregated. The IDPs are known to be involved in numerous interactions with multiple partners. Therefore, they frequently serve as nodes or hubs in protein interaction networks, where they are central to the normal function and stability of the network [16]. αSyn is a typical IDP, in fact adopting different conformational states and interacting with more than 50 others proteins [17], interactions that, in turn, may cause changes in αSyn conformation [18,19]. It is still not known if the multitude of binding partners competes for similar binding sites, interacts differentially according to Syn conformation or binds in a competitive/allosteric binding and if these bindings take place in specific subcellular compartments. Although αSyn’s physiological role is unknown, some evidence suggests a potential implication in the exocytic process [20], in the recycling of synaptic vesicles [21] and in the regulation of synaptic transmission [22].

## 2. αSyn Pathological Species

### 2.1. αSyn Aggregation

In the Lewy bodies, αSyn is found in fibrillar aggregates [23], and some evidence indicates that the oligomeric and fibrillar αSyn species are responsible for the toxicity and the spread of neurodegenerative diseases [24]. The mechanism involved in αSyn oligomerization seems to be influenced by different factors, such as the levels of αSyn expression [25], the form of αSyn (wild-type or mutated) [26], pH and temperature [18,27], the concentration of some metals (*i.e.*, aluminum) [28], and also by environmental agents, like pesticides [29]. In the Lewy bodies, αSyn adopts a β-sheet structure that is highly organized. *In vitro* studies showed that the oligomerization process is nucleation dependent, starting from αSyn monomers or dimers to the anti-parallel β-sheet [30]. Initially, the protein changes its conformation from unfolded to partially folded oligomers. This conformational change permits the exposure of the NAC domain that starts the aggregation process through hydrophobic interactions. Monomers are rapidly added to the initial nuclei with the formation of large oligomers, protofibrils and, at last, fibrils [18]. Cellular processes altered by the presence of fibrillar αSyn species whose impairment leads to neuronal toxicity and death and are well studied: reduction in the size of the presynaptic vesicular pool [31], mitochondrial dysfunction [32], increase of the level of intracellular reactive oxygen species [33], formation of pores in the plasma membrane [34,35] and the inhibition of the ubiquitin-proteasome system [36].

### 2.2. αSyn Mutations

The αSyn gene, *SNCA*, is located on chromosome 4q22.1 and contains six exons encoding the 140-amino acid of the protein. The first genetic evidence for the involvement of *SNCA* in PD was the identification of three missense mutations (A30P, E46K and A53T), which segregated with the disease in unrelated families and caused PD with high penetrance [37,38,39]. Up to now, another two mutations (H50Q, G51D) have been discovered in the αSyn gene [40,41,42,43].

The different mutations exhibit distinct effects on the rate of progression of PD. For example, patients carrying the A30P mutation show a late and mild form of dementia, whereas carriers of the A53T mutation are affected by a severe form of parkinsonism frequently associated with dementia [38,44]. Moreover, Conway *et al.* showed that A53T αSyn *in vitro* fibrillizes more rapidly than the wild-type form, while A30P αSyn fibrillizes more slowly [45]. H50Q mutation has been shown to increase the rate of αSyn aggregation, whereas the G51D mutation has the opposite effect, despite the early onset of disease in PD patients bearing the G51D mutation. Indeed, both αSyn mutants can form intracellular aggregates starting from internalized amyloidogenic preformed seeds [46]. Jensen *et al.* were the first to show that the A53T form, unlike the A30P form, maintains the ability to bind vesicles and membranes [4]. However, in some conditions, the different mutations have the same pathological effect. Tanaka *et al.* using an *in vitro* cell culture demonstrated that the expression of the A30P form leads to a decreased activity of the proteasome complex after seven days [47]. Nonaka and Hasegawa showed a similar inhibition of the proteasome activity in SH-SY5Y expressing A53T αSyn. The ability of the mutant forms of αSyn to inhibit the proteasome activity is probably related to the propensity of αSyn to assemble into filaments [48]. Additionally, the expression of both A30P and A53T αSyn make the cells more vulnerable to oxidative stress [49,50] or dopamine toxicity [51].

### 2.3. αSyn Dosage

Although mutations in the αSyn gene have been implicated in the progression of PD, recent genetic and biochemical data suggest that an increase in the level of the expression of wild-type αSyn is sufficient to cause neurodegeneration [52]. Ross *et al.* in their work showed that patients with *SNCA* duplications often exhibit a classical PD phenotype. On the other hand, the more rare cases of triplications exhibit a more severe phenotype, showing a direct relationship between *SNCA* gene dosage and pathology [53]. Devine *et al.* in their work used induced pluripotent stem cells (iPSCs) derived from patients with triplication of the *SNCA* locus. These patients display PD symptoms, and their iPSCs differentiated into dopaminergic neurons express double the amount of αSyn. The increase in αSyn expression might be responsible of the disease in these individuals [54]. In another study, Flierl *et al.* demonstrated that iPSCs with *SNCA* triplication, once differentiated into the neuronal precursor, exhibit normal cellular morphology, but showed changes in growth, viability and stress resistance. More importantly, αSyn silencing by shRNA rescues these phenotypes [55].

αSyn levels may also have a role in the pathogenesis of sporadic PD; nucleotide polymorphisms, highly associated with PD and affecting αSyn levels by altering gene transcription or mRNA stability, were recently identified. Moreover, genetic variations in the promoter region, in the REP1 dinucleotide repeat, previously found to regulate *Syn* expression, appear to modulate PD risk [56,57]. Interestingly, the selective vulnerability of dopaminergic neurons in the substantia nigra with respect to the neurons of the ventral tegmental area may correlate with the increased Syn levels found in these neurons in aged monkeys and humans [58].

### 2.4. αSyn Post-Translational Modifications

αSyn undergoes several post-translational modifications, which have a potential role in αSyn aggregation and toxicity. Phosphorylated [13], Tyr-nitrated [12] and methionine-oxidized [59,60] products of αSyn are found in Lewy bodies, as well as truncated forms of the protein, obtained by calpain I cleavage [61]. αSyn is constitutively phosphorylated at various serine residues, with serine 129 (S129) as the major phosphorylation site [62]. Numerous studies showed that S129 is phosphorylated in more than 90% of the insoluble αSyn present in Lewy bodies, whereas the phosphorylation is present only in 4% of normal αSyn [13]. Many authors identified different kinases mediating αSyn phosphorylation at S129. Okochi *et al*. produced evidence that S129 is phosphorylated by casein kinase 1 and casein kinase 2 [62]. Pronin *et al.* in their study demonstrated that αSyn is a substrate of G protein-coupled receptor kinases (GRK). Moreover they showed that GRK-mediated phosphorylation inhibits the interaction of αSyn with phospholipids and phospholipase D2, suggesting a functional role for this specific modification [63]. The function of this post-translational modification is not completely clear. Waxman and Giasson suggest that phosphorylation in S129 blocks αSyn fibrillization [64]. Chen and Feany using the *Drosophila* model of PD showed that mutation of S129 to alanine to prevent phosphorylation completely suppresses dopaminergic neuronal loss produced by expression of human αSyn. Consistently, mutation of S129 with the negative residue aspartate, which mimics phosphorylation, increases αSyn toxicity [65]. Moreover Visanji *et al.* demonstrate that the phosphorylation state of S129 can influence the ability of αSyn to bind synaptic membrane [66]. Recently, also, phosphorylation in serine 86 (S86) was characterized. Paleologou *et al.* suggest in their study that phosphorylation at S86 maintains αSyn as unfolded, blocks its fibrillization *in vitro* and reduces the binding of αSyn to membranes [67]. It is has been shown that αSyn purified from Lewy bodies is partially ubiquitinated [68]. Ubiquitination is a process that controls numerous aspects of protein function, such as degradation, protein-protein interaction and subcellular localization [69]. Nonaka *et al.* report that *in vivo* αSyn is ubiquitinated at lysine 6, 10 and 12. Moreover, they showed that fibrils of αSyn are less ubiquitinated than the soluble form and suggested that de-ubiquitination may have implications for the formation of αSyn toxic forms [70]. In recent studies, quite a few aggregation-prone proteins implicated in neurodegeneration were found to be SUMOylated, and SUMOylation-deficient mutants showed an enhanced tendency to aggregate [71]. Krumova *et al.* showed that SUMOylation of αSyn is able to delay protein aggregation [72]. Furthermore, Kunadt and collaborators proposed that SUMOylation of αSyn is directly involved in the release of the protein in the extracellular space [73].

## 3. Physiopathological Role of αSyn in Neurosecretion

Considering that the physio/pathology of αSyn is strictly dependent on its level of expression and ensuing propensity to aggregate, it is fundamental to study the anatomical and ultra-structural distribution of αSyn. αSyn is expressed in many types of cells, both neuronal and non-neuronal. Indeed, αSyn is especially expressed in hippocampus, olfactory bulb, striatum and cerebellum [74,75], but also in the peripheral nervous system at the level of the olfactory system, retina and auditory system [76]. From the point of view of neuronal specificity, αSyn is not exclusively expressed in monoaminergic neurons of the CNS, as first described in [77], but it is also ubiquitously expressed in cholinergic, GABAergic and glutamatergic neurons [78]. αSyn was detected also in other cell types than neurons, such as astrocytes [79], oligodendrocytes [80], macrophages [81], endothelial cells [82] and platelets [83]. Regarding its localization in neurons, αSyn was first identified in association with synaptic vesicles and in the nucleus [1], where it binds histones [84,85], and only later the subcellular fractionation of mouse brain revealed αSyn presence in the cell body and in neurites [86]. The localization of αSyn in neuronal cell bodies early in development followed by a redistribution to the nerve terminals, suggests that αSyn is involved in synaptogenesis [87]. The delayed localization at synapses is consistent with the idea that αSyn may function in the maintenance, rather than in the formation, of synapses [31]. The function of αSyn is not clear, and the mechanism by which it leads to pathology is poorly understood. Based on the findings of αSyn enrichment at the presynaptic compartment and of its loose association with synaptic vesicles, a role for this protein in neural transmission has been hypothesized [88,89]. Since its identification, αSyn has been demonstrated to be involved in synaptic plasticity [90]: upregulation of αSyn has been associated with the process of learning in experimental animals, such as song-learning in birds [91,92]. Even though αSyn is highly soluble, it binds to a diversity of cellular membranes and proteins with different properties. These interactions are considered critical for the physiological function of αSyn [93].

### 3.1. Synaptic Vesicles and Transmitter Release

αSyn’s role in synaptic vesicles homeostasis is well characterized, and a large number of studies point out αSyn’s involvement in every phase of the synaptic vesicle cycle, including trafficking, docking, fusion and recycling after exocytosis. Murphy *et al.* demonstrated that a reduction in αSyn expression after treatment of a neuronal cell culture with antisense oligonucleotide led to a reduction in the size of the presynaptic vesicular resting pool [94]. Abeliovich *et al.* established a αSyn knock-out mouse model. They showed that αSyn^−/−^ mice are viable and with no changes in brain architecture. Moreover, dopaminergic neurons are healthy and do not present anomalies in the release or uptake of dopamine. However, these mice display an incremented release of dopamine upon paired pulse stimulation and ensuing altered dopamine-dependent locomotor response to amphetamine [22]. These works suggest that αSyn is a negative presynaptic regulator of neurotransmitter release that would restrict the traffic of synaptic vesicles from the resting pool to the sites of release. Consistently, it was shown that an increase in the refilling of the ready releasable pool of vesicles in the striatum of αSyn^−/−^ mice or of mice expressing the A30P αSyn mutated form [95] also showed a reduced size of the resting pool [96]. This kind of negative regulation appears not confined only to dopamine release. Experiments with transgenic mice overexpressing A30P αSyn or with different strains of αSyn knock-out mice revealed an altered norepinephrine mobilization [97]. Furthermore, other studies suggested the idea that the lack of αSyn also impairs the mobilization of glutamate from the reserve pool and that, on the contrary, the expression of the A30P mutated form of αSyn does not exhibit this effect [98]. Experiments of αSyn overexpression link this protein also to vesicle trafficking. In PC12 cells, the overexpression of wild-type αSyn causes the accumulation of docked vesicles, suggesting an inhibition of the priming of neurosecretory vesicles, which are unable to fuse [99]. In hippocampal neurons, it has been shown that the overexpression of αSyn impairs the re-clustering of synaptic vesicles after endocytosis [100].

Another αSyn-null mouse yielded results apparently inconsistent with the above interpretation. In their mouse model, Cabin and collaborators [101] showed that the absence of αSyn correlated with an impaired response to tetanic stimulation, due to deficient refilling of the ready releasable pool, possibly due to a reduction in the size of the resting pool. The further characterization of a double knock-out mouse model, lacking both the α- and β-isoform of the synuclein family, evidenced no impairment in basic brain functions or survival, with no alteration in synapse morphology, in synaptic plasticity, in the size of synaptic vesicle pools or in synaptic vesicle recycling. Differently from the single αSyn knock-out model, double knock-out mice showed a characteristic decrease of dopamine levels in the brain, with no change in uptake and release. These data suggest that the synuclein family is not an essential component of the synaptic machinery, but may contribute to more subtle long-term regulation and/or maintenance of function at the presynapse [102]. Recently, data about a triple-knock out mouse lacking the three α-, β- and γ-isoforms of synuclein have been reported. These mice showed altered synaptic structure and transmission, age-dependent neuronal dysfunction and diminished survival. In particular, a 30% decrease of the size of excitatory synapses was observed. A further characterization of triple knock-out mice evidenced a non-correct formation of the N-ethylmaleimide-sensitive factor (NSF) attachment proteins (SNAPs) receptor family of proteins (SNAREs) complex [103,104]. In particular, αSyn binds to synaptobrevin2/vesicle-associated membrane protein 2 (VAMP2), thus promoting SNARE’s assembly. The hypothesis that αSyn acts to support SNARE complex assembly and functioning during aging seems in accordance with its putative physiological role at presynaptic terminals.

Finally, some other studies sustained that αSyn may positively regulate synaptic transmission, by acting on the late steps of exocytosis. In cysteine string protein alpha (CSPα) knock-out mice, transgenic expression of αSyn was shown to rescue the function of SNAREs, abolishing the lethality that takes place in these mice [20]. In hippocampal neurons, the introduction of αSyn enhanced both spontaneous and evoked neurotransmitter release, while its deletion by antisense oligonucleotides blocked the potentiation of synaptic transmission [105]. In knock-out animals, in the absence of αSyn was reduced the frequency and amplitude of synaptic currents in neurons of area CA1 of the hippocampus, but only in highly demanding conditions, when the release probability was high [106].

Lundblad and collaborators used *in vivo* amperometry to monitor changes in synaptic dopamine release in the striatum induced by the injection of an adeno-associated virus-type vector carrying αSyn in nigral dopaminergic neurons. They showed that the impairment in dopamine release correlated with damage in nigrostriatal axons and terminals. After three weeks from the injection, the first signs of axonal damage were visible, and the amount of dopamine released after a KCl pulse was reduced by 70%–80%. After 8–16 weeks, the overall striatal innervation density was reduced by 60%–80%, and the presence of αSyn aggregates was shown. At this stage, dopamine release was reduced by 80%–90% [107]. Several mechanisms have been proposed regarding the possible role of αSyn in the regulation of vesicles trafficking. Ahn *et al.* in their study demonstrated that αSyn is capable of binding phospholipase D2 (PLD2) and inhibiting its activity [108]. PLD2 catalyzes the hydrolysis of phosphatidylcholine to generate the lipid second messenger, phosphatidate and choline and is implicated in the regulation of vesicle trafficking in terms of endo- and exo-cytosis [109]. Moreover αSyn can indirectly inhibit PLD2 through its binding to proteins that regulate PLD2 activity, such as extracellular signal-regulated kinase (ERK) or 14-3-3 protein [110]. Another key player in the regulation of neurotransmitter release is the actin cytoskeleton. Actin microfilaments are essential for synaptic vesicle mobilization between different functional pools, for their organization at the active zone, thus influencing the exocytotic process. Actin has been suggested to act via multiple mechanisms. Actin microfilaments could arrange tracks along which the vesicles travel or bring the vesicles on top of the waves of polymerization. Actin filaments could also act as a physical barrier opposing exocytosis. Moreover, actin is required to cluster the molecular complex essential for vesicles fusion. Recent studies suggested that αSyn binds actin, modulating its polymerization in a manner dependent on Ca^2+^ concentration [111]. A30P αSyn alters the rate of actin polymerization and perturbs cytoskeleton morphology and dynamics, leading to alterations in the exo-/endo-cytic traffic [112].

### 3.2. Calcium Entry

Ca^2+^ is a highly versatile intracellular signal that regulates many different cellular processes and plays a pivotal role in neuronal plasticity and survival [113]. The network that regulates intracellular calcium level is extremely complex, and Ca^2+^ signals depend on its influx from the extracellular space together with its release from intracellular stores, such as the endoplasmic reticulum [114]. Neurons express a multitude of Ca^2+^-binding proteins. Ca^2+^ sensors decode and differentiate between various Ca^2+^ signals according to differences in the localization, Ca^2+^ affinity and kinetics of ion binding [115]. Ca^2+^ clearance mechanisms in neurons control the duration, as well as the spread of Ca^2+^ signals and result in the reduction of free cytoplasmic Ca^2+^ and in the restoration of its basal level during recovery from stimulation [116]. An abnormally-increased Ca^2+^ concentration demands intense Ca^2+^ buffering, a demand that is not met by aging neurons. Rapid Ca^2+^ sequestration is attributed to Ca^2+^-binding protein buffers in the cytoplasm, such as calbindin, calretinin and parvalbumin.

Recently, deregulation of this network has been correlated with neurodegeneration occurring in sporadic Parkinson’s disease (PD) [117]. Surmeier *et al.* showed that epidemiological data support a linkage between Ca^2+^ channels and the risk of developing PD. The main dopaminergic midbrain subpopulation affected in PD is a subpopulation of ‘A9’ nigrostriatal neurons, while neurons, including the ‘A10’ dopaminergic neurons of the ventral tegmental area (VTA), are largely spared. Dopaminergic neurons expressing higher levels of protein buffers calbindin, calretinin and parvalbumin seem to be resistant to degeneration in PD [118,119,120] and in the 1-methyl-4-phenyl-1,2,3,6-tetrahydropyridine (MPTP)-treated mouse model [121]. Moreover, selective vulnerability of *substantia nigra pars compacta* (SNc) dopamine neurons to aging-related Ca^2+^ dyshomeostasis has recently been supported by the observation that pacemaker firing in these cells relies on L-type voltage-operated Ca^2+^ channels (VOCCs), as opposed to juvenile cells, which rely on Na^+^ channels. Prolonged activity of L-type VOCCs in older neurons may lead to Ca^2+^ overload and contribute to neuronal ageing and death. Moreover, they pointed out that L-type VOCCs elevate the sensitivity of dopamine neurons to mitochondrial toxins used to create animal models of PD, suggesting that Ca^2+^ entry is a key factor in their selective vulnerability [122].

Studies on the effect of αSyn on Ca^2+^ homeostasis have been mainly conducted by using αSyn oligomers. αSyn aggregates potentiate neuronal Ca^2+^ dyshomeostasis and overload [123,124,125]. Various works showed an increase of Ca^2+^ in basal conditions, possibly due to a pore-forming mechanism [125], and an alteration in membrane conductance due to leak channel formation [126], consistent with *in vitro* evidence demonstrating the formation of pore-like structure in synthetic membranes [127].

Furukawa *et al.* reported that in a neuroblastoma cell line, the expression of both A30P and A53T mutant forms of αSyn increases the plasma membrane ion permeability. Both the basal level of Ca^2+^ and the level after membrane depolarization are greater in cells expressing mutant of αSyn, and the Ca^2+^ chelator 1,2-bis(2-aminophenoxy)ethane-N,N,N',N'-tetraacetic acid (BAPTA-AM) significantly protects the cells from oxidative stress [128]. In the SH-SY5Y cell line expressing wt or the A53T mutant αSyn, Ca^2+^ entry through L-type VOCCs was increased, and store-operated Ca^2+^ entry (SOCE) following store depletion was suppressed [129]. Martin and collaborators in their study demonstrated that αSyn oligomers can increase intracellular Ca^2+^ levels, inducing calcineurin activity, which leads to death of human neuroblastoma cells. They also showed a consistent effect when αSyn oligomers were applied to organotypic brain slices or in mice after acute intracerebroventricular injections [130].

### 3.3. Membranes

Due to its lack of transmembrane domain or lipid anchor, Syn has been considered a peripheral membrane protein. However, multiple studies led to the observation that Syn is able to bind phospholipidic membrane and free fatty acids, both *in vitro* and *in vivo*. It is well known that *in vitro* αSyn binds artificial membrane, in particular those containing acidic phospholipids. The N-terminal region of αSyn is able to bind synthetic lipid vesicles and to detergent micelles *in vitro* and mediates its transition from random coil to an α-helical structure, while the C-terminal part of the protein does not associate with either vesicles or micelles, remaining unstructured [89,131,132,133,134]. The missense mutations linked to familial forms of PD occur at the N-terminal domain of αSyn. Unexpectedly, both A30P and A53T αSyn do not significantly affect the structure of membrane-associated αSyn, while E46K shows an increase in αSyn affinity for an artificial membrane [135]. Interaction of αSyn with membranes has been investigated *in vivo* by several groups. αSyn interacts with membranes at the synapse, but in a transient, rapid and reversible manner [136]. On the contrary, the A30P mutation greatly diminishes the propensity of Syn to bind to native membranes [4,137,138]. Fortin *et al.* demonstrated that αSyn associates specifically with lipid microdomains resistant to low-temperature detergent solubilization, the lipid rafts [139]. Importantly, they showed that the A30P mutant lost the ability to interact with synaptosomal membranes and redistributed away from the synapse. This suggests a role for raft association in the physiological function of the protein [140]. Neuronal activity controls both the synaptic localization and membrane association of αSyn. Upon the entry of Ca^2+^ that triggers the exocytic process, αSyn rapidly disperses away from synaptic boutons, dissociating from synaptic vesicles membrane after exocytosis [136].

The modulation performed by αSyn on synaptic vesicle recycling may also be related to a further role of the protein in the regulation of fatty acid metabolism [141,142,143]. αSyn regulates the overall level of polyunsaturated fatty acids and modulates the activity of enzymes, such as acyl-CoA synthetase, which are critical in the fatty acid re-acylation pathway [144,145].

Kamp and Beyer in their work entered more in detail and, using electron spin resonance (ESR) spectroscopy, showed that binding of αSyn to cholesterol- and sphingomyelin-containing vesicles could help in their stabilization, protecting them from premature fusion [146]. Dikiy and Eliezer presented a model in which αSyn acts as a bridge with its N-terminal region bound to the plasma membrane and its C-terminal domain bound to docked or budding vesicles [147]. Membrane interactions have also been implicated in αSyn aggregation. In particular, Lee and collaborators demonstrated that, when recombinant αSyn was added to brain membranes, the rate of aggregation of the fibrillization rate of the protein was increased [148].

## 4. Extracellular αSyn

Even if αSyn has been considered exclusively an intracellular protein, recently, this idea was challenged by the discovery of αSyn in the cerebrospinal fluid and blood in both normal and PD subjects [149,150]. The data collected so far did not give consistent results to address the question of whether the level of extracellular αSyn might be considered a reliable biomarker for αSyn-pathology. αSyn levels were reported to be significantly increased in patients affected by PD and multiple system atrophy [151], while in a different study, αSyn extracellular level appeared lower in PD-patients than in control subjects [152]. One interpretation of PD etiopathogenesis seemed to rely on a causative agent transmitted via retrograde and transneuronal transport to the susceptible brain regions, from enteric nerves, to lower brain stem nuclei, then to the midbrain and, finally, to cortical areas [153,154]. Increasing evidence supports the idea that αSyn might actually act as the pathogen responsible for the spreading of neurodegeneration. The mechanism of αSyn secretion from cells remains largely unknown, and the more interesting question is how endogenous αSyn is released in the extracellular space.

### 4.1. αSyn Release

To assess how αSyn is released from neurons, Lee *et al.* overexpressed human αSyn in neuroblastoma cells and in rat primary cortical neurons using an adenoviral vector. They demonstrated the presence of αSyn in the culture medium after two hours and an accumulation over time, suggesting a process of constitutive release from cells. To determine if αSyn release is mediated by exocytosis, they used incubation at low temperature, a blocker of vesicular exocytosis. They found that low temperature reduces the secretion of the protein, suggesting that αSyn is released from the cells by exocytosis in a stimulation-independent manner. Moreover, they used brefeldin A (BFA), an inhibitor of the classical ER-Golgi pathway [155,156], showing that BFA does not alter the release of αSyn, which thus appears to be independent of the ER-Golgi pathway. Furthermore, they demonstrated the presence of αSyn in the lumen of the vesicles and that aggregation of αSyn is favored in vesicle lumen [25]. The ER-Golgi pathway for secretion seems to be involved in others regions of the nervous system. In fact, Paillusson *et al.*, working on a primary culture derived from the enteric nervous system (ENS), showed a level of secreted αSyn similar to the one present in other biological fluids [157]. The treatment of this primary culture of ENS with BFA drastically reduces the secretion of αSyn, suggesting that in the enteric neurons, the conventional pathway plays a central role [158]. Emmanouilidou *et al.* demonstrated that αSyn is physiologically secreted, associated with membrane vesicles that are similar in size, morphology and protein composition to exosomes. Moreover, using Ca^2+^ ionophores/chelators, they found that αSyn secretion is affected by intracellular calcium concentration [159]. Upon mitochondrial or proteasomal inhibition, neurons were found more prone to release both monomeric and aggregated αSyn, arguing in favor of the possibility that αSyn is secreted upon defects in its folding or processing, due to conditions of cell stress [25]. To better understand the pathway involved in αSyn secretion, Hasegawa *et al.* [160] analyzed the multivesicular bodies (MVBs). MVBs are endocytic organelles with two destinations: they are responsible for the sequestration of proteins that are condemned to lysosomal degradation, and they undergo exocytic fusion with the plasma membrane, which leads to the release of intraluminal vesicles into the extracellular environment [161]. Vacuolar protein sorting 4 (VPS4) is a key component of the MBV exocytic pathway [162]. Using a dominant negative VPS4A in αSyn-expressing HEK293T and SH-SY5Y, Hasegawa *et al.* showed an increase in extracellular αSyn, suggesting that a perturbation in MVB-exosome genesis affects the process of αSyn release [160].

### 4.2. αSyn Internalization

**Figure 2:**
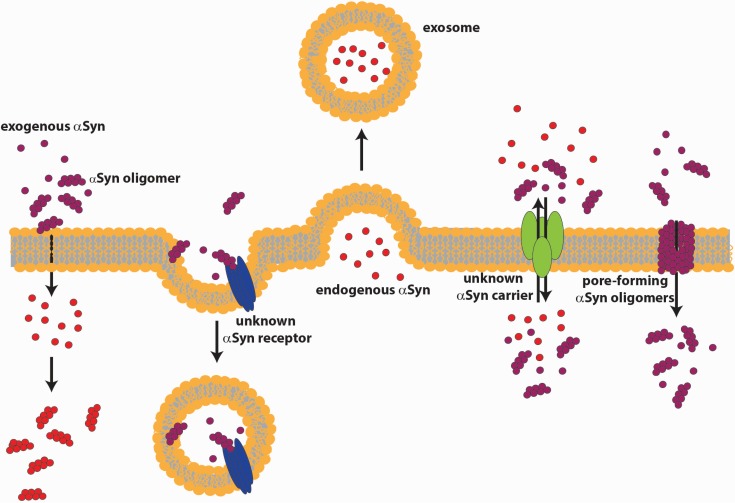
Mechanisms of αSyn release and internalization. αSyn can be released from healthy neurons by conventional exocytosis of vesicles or MVB, or through exosomes, or can pass the membrane with the help of an unknown carrier. αSyn can enter neurons by internalization in vesicles, or through pores formed into the membrane by αSyn oligomers, or by direct translocation across the plasma membrane.

The question of how extracellular αSyn in both the monomeric and oligomeric form contributes to neuronal toxicity in PD has been the subject of intensive research. Extracellular proteolytic enzymes, as matrix metalloproteases, were shown to degrade αSyn with the production of smaller protein species highly prone to aggregation [163]. Besides being extracellularly degraded, αSyn might be internalized by neurons [164], microglia [165,166] and astrocytes [167]. Many studies focused on the mechanism of αSyn uptake into cells. One hypothesis suggests the ability of αSyn oligomers to form pentameric pore-like structures in cell membranes that increase intracellular calcium, leading to oxidative stress, lysosomal leakage and mitochondrial dysfunction, resulting in cell vulnerability and neurodegeneration [168]. Feng *et al.* used a dopaminergic-like cell model with regulated αSyn expression, reporting an increase in membrane permeability and conductance due to pore formation. More importantly, they show for the first time that the extracellular application of an anti-αSyn antibody reverts the effects on membrane permeability, suggesting an αSyn interaction with the outer surface of the cell membrane [126]. Danzer *et al.* entered more in detail about αSyn species and showed that different aggregation conditions produce heterogeneous populations of αSyn oligomers, which can be differentiated on the basis of their biophysical properties and cellular effects. SH-SY5Y cells treated with Type A oligomers induced an increased membrane permeability and triggered cell death, while Type B and C oligomers were able to enter cells directly and to seed intracellular αSyn aggregation [125]. Moreover, they demonstrated that the Type C oligomers are capable of inducing transmembrane αSyn seeding in a dose- and time-dependent manner, also in cortical primary neurons [169]. Lee *et al.* showed that the internalization of αSyn aggregates in cells is inhibited by the expression of a dominant-negative dynamin-1 or by low temperature, indicating that the internalization depends on endocytosis. More in depth, they demonstrated that this form of endocytosis of αSyn aggregates depends on an unknown membrane surface receptor [170]. Many authors suggest that monomeric αSyn internalization occurs very rapidly via a mechanism distinct from normal endocytosis. In fact, they show that the protein is detectable in the cytoplasm of the cells after five minutes of incubation. Moreover, the import of αSyn is not affected by temperature or the inhibitor of endocytosis, suggesting a direct translocation across the plasma membrane [167,170]. These distinctions were partially contradicted by the very recent finding that neuron-to-neuron transfer of monomeric, oligomeric, as well as fibrillar αSyn relied on endocytic processes, as demonstrated by experiments performed with selective endocytosis inhibitors, both *in vitro* and *in vivo* [171]. Sung *et al.* suggest that αSyn alone is not able to traverse the membrane and identified a possible carrier with a 60-kDa molecular size, which appears to bind to αSyn in a specific way [164]. Membrane trafficking plays a central role in the maintenance of cell organization and organelle homeostasis and is necessary for intercellular signaling [172]. Chai *et al.* in their work used transferrin-mediated iron uptake [173] to study alteration in intracellular trafficking induced by αSyn oligomers. They show that after internalization of the oligomers, the rate of transferrin receptor recycling is increased, and consequently, the surface expression of the receptor is modified. [174]. It has been suggested that microglial inflammation augments the progression of PD [175]. Using different primary mesencephalic cultures, Zhang *et al.* demonstrated that αSyn aggregates can be phagocytized into microglia cells. Subsequent activation of NADPH oxidase plays a central role in microglial activation of the inflammation process, leading to neurotoxicity [165]. Similar results were obtained by performing experiments with primary astrocytic cultures or astrocytoma cell lines, which exhibited the acquisition of a reactive phenotype upon incubation with extracellular αSyn [176,177]. The toxic phenotype observed in neurons seems to derive from the non-cell autonomous interaction between neurons and glia, mediated by αSyn, which may lead to chronic inflammation. It has to be reminded that both PD-affected patients and animal models show signs of chronic inflammation. Since αSyn seems to be implicated in exocytosis [20] and in the recycling of the synaptic vesicles [21], accumulation of αSyn monomers as a result of constant internalization could also alter the physiological state of membrane trafficking and synaptic transmission. The model in Figure 2 depicts various possible routes of release and internalization of αSyn.

### 4.3. Extracellular αSyn Action

Some data sustained that αSyn, after being internalized, may also trigger the seeding of endogenous proteins, both in neuroblastoma cells and in primary cortical neurons [125,169]. Even though in earlier studies, αSyn-mediated seeding occurred only with the assistance of artificial techniques, as liposomes, [64,178], a more recent work showed that this process might occur even with only the presence of extracellular αSyn *per se*, either in its monomeric or polymerized structure [171]. A report showed the presence of αSyn-containing depositions in proliferating stem cells engrafted in the hippocampus of a PD-mouse model [179]. Finally, a recent study mimicked more closely the clinical situation for human patients, by grafting fetal dopaminergic neurons, at a postmitotic developmental stage, into the striatum of mice overexpressing human αSyn, showing the transfer of human Syn to grafted cells, where it seeded the assembly of soluble αSyn, thus forming toxic deposits [171]. Another study provided evidence for the transfer of αSyn from an intrastriatal inoculation to recipient cells, where it seeds the assembly of soluble αSyn, leading to Lewy pathology in a connectivity-dependent way, propagating along interneuronal circuits [180]. In a very recent work, injection of a non-amyloidogenic, a truncated form of αSyn, was shown to induce neuronal pathology, such as dystrophic neurites and astrogliosis, eight month after injection, indicating that pathological alterations cannot be attributed only to conformational-dependent templating events and pointing to the importance of a high dosage of soluble αSyn in the onset of the disease. Notably, besides the increase in αSyn expression due to duplication and triplication of the αSyn gene, any form of brain injury may promote the release of monomeric cellular αSyn; also, αSyn release could occur during neurodegeneration upon neuronal death [181].

In PD models of αSyn overexpression, dopaminergic neuron loss is preceded by degenerative changes in striatal axons and terminals, indicating that αSyn-induced pathology hits the axons and terminals first and suggesting that the cell bodies are involved by a dying back mechanism.

**Figure 3:**
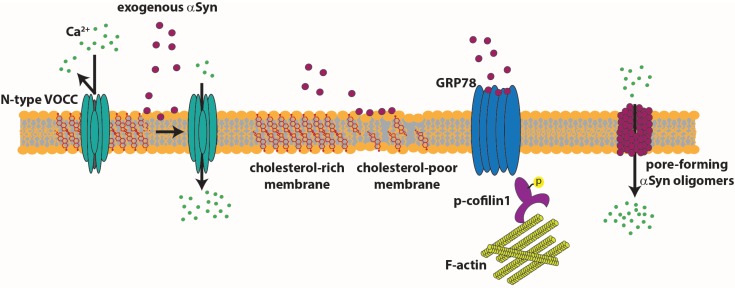
Targets of extracellular αSyn. Extracellular soluble αSyn induces an increase in calcium entry by acting on the fluidity of the membrane or by activating N-type calcium channels that shift to cholesterol-poor domains of the membrane. Extracellular soluble αSyn clusters GRP78 at the plasma membrane, with ensuing activation of a pathway that leads to cofilin 1 phosphorylation.

αSyn monomers and oligomers might also exert their effect directly from the extracellular space on pre- and post-synaptic terminals. Diogenes *et al.* showed that, in rat hippocampal slices, prolonged exposure to αSyn oligomers determines an increase of basal synaptic transmission dependent on N-methyl-D-aspartate (NMDA) receptor activation. Moreover, they showed that the increase in NMDA receptor activity triggers an enhanced contribution of GluR1-containg α-amino-3-hydroxy-5-methyl-4-isoxazolepropionic acid (AMPA) receptor. In the long term, this non-physiological activity of AMPA receptors leads to impaired long-term potentiation (LTP) [182]. Melachroinou *et al.* used the application of naturally-secreted αSyn on rat cortical neurons and showed that extracellular αSyn can perturbs Ca^2+^ homeostasis with a mechanism that alters the fluidity property of the membrane [183]. Ronzitti and collaborators demonstrated that dysregulation of neurotransmitter release induced by monomeric extracellular αSyn depends on the activation of surface-exposed calcium channels. They provided evidence that extracellular αSyn, applied on rat cortical neurons or striatal slices, selectively activates N-type VOCCs, inducing neurotransmitter release. Moreover, they correlated the effect of αSyn with the reduction of membrane cholesterol and with the ensuing alteration in partitioning of N-type VOCCs, which move from raft to cholesterol-poor areas of the plasma membrane [184]. αSyn’s role in the regulation of axonal transport of synaptic vesicles [185] and of synaptic vesicle mobilization at the terminal might be mediated by a specific binding with proteins belonging to or associated with microtubules and microfilaments. Syn may interact with the MT-binding domain of protein tau [186], MT-associated protein 1B (MAP1B) [187] and 2 (MAP2) [188]. αSyn and actin has been observed to partially co-localize in neuronal cell lines [189]. αSyn can also influence the cytoskeleton of cultured neurons when applied to the extracellular milieu. Using a biochemical approach, Alim *et al.* showed that αSyn binds to heterodimeric tubulin and may seed αSyn fibril formation, an interaction capability that is lost by the mutant forms of αSyn [190]. Liu and collaborators demonstrated that application of wt αSyn to cultured primary rat cortical neurons leads to microtubule assembly and neurite outgrowth. The A30P and the A53T mutant forms do not show the same effect [191]. Various studies indicated a strong relationship between αSyn and the microtubule-associated protein tau in the neurodegenerative process [192,193]. The major function of tau is the stabilization of microtubule dynamics necessary for neurite outgrowth, morphogenesis, axonal transport and physiological neuronal function [194]. Gassowska *et al.* suggest that the interaction of αSyn with tau, increasing phosphorylation of tau by GSK-3β, leads to microtubule destabilization [195]. Bellani *et al.* showed that the interaction of αSyn with a surface-exposed glucose-related protein of 78 kDa (GRP78) activates a signaling cascade that, acting on cofilin 1, affects the morphology and dynamics of the actin cytoskeleton. Downregulation of GRP78 abolishes the activity of exogenous αSyn, suggesting that it is the primary target of αSyn [196]. In the model in Figure 3 are shown the targets of extracellular αSyn acting from the extracellular space.

## 5. Conclusions

The studies reported here highlight the multitude of possible pathological effects of αSyn. This relevance of αSyn has stimulated the development of numerous animal models obtained with genetic approaches (deletion or overexpression of the *SNCA* gene) or chemical perturbation. Animal models have obvious limitations in representing the overall features of the pathology, in that very few of them recapitulate the characteristic signs of PD, *i.e.*, consistent neuronal damage in the nigrostriatal pathway and the formation of Lewy bodies. However, they offer invaluable insight for the analysis of the specific mechanisms of PD pathogenesis.

Models alternative to mice have been used, either because they allow easier genetic manipulation and large-scale screening for the discovery of novel pathways involved in the pathology or, on the other side, because their neuronal circuits more closely resemble that of humans. Lakso *et al.* overexpressed both wt and A53T αSyn by injection into *Caenorhabditis elegans* and obtained neuronal and dendritic loss and motor deficits [197]. Transgenic *Drosophila* expressing wt, A30P or A53T αSyn replicate the features of human PD, with dopaminergic neuron loss, filamentous intraneuronal αSyn inclusions and locomotor dysfunction [198]. Yamada *et al.* used a recombinant adeno-associated viral vector system for human αSyn gene transfer to rat substantia nigra and observed approximately 50% loss of dopaminergic neurons at 13 weeks after infection [199]. Overexpression of human αSyn in rats using a human BAC construct containing the entire *SNCA* sequence led to neurotoxic conversion of monomeric αSyn into insoluble aggregates in striatum, severe loss of dopaminergic integrity and a behavior phenotype similar to that in human PD [200]. Moreover rat lentiviral-based models of PD expressing A30P or A53T αSyn exhibit protein aggregates, selective loss of nigral dopaminergic neurons and α-synucleinopathy [201]. Transgenic monkeys overexpressing wt or A53T αSyn developed motor impairments, αSyn-positive inclusions and dystrophic neurites, resembling the chronic and progressive characteristics of human PD [202].

In mice, the expression of wt and mutant forms of αSyn causes effects frequently observed in human PD. Van der Putten and collaborators showed that expression of wt and A53T αSyn in the nervous system of mice generated animals with neuronal α-synucleinopathy, neuronal degeneration and motor defects [203]. Similarly, the overexpression of A30P αSyn led to motor dysfunction, reduced size of the dopamine (DA) storage pool, decreased locomotion and impaired motor coordination and balance [96,204].

Between all of the models, it appears that lentiviral injection for αSyn expression induces a more severe phenotype and dopaminergic neuronal death, possibly because of a lack of compensatory mechanisms, as might happen in transgenic animals. Thus, the construction of novel animal models with conditional expression of αSyn, as well as of other members of the synuclein family, regulated for time and localization of expression, may provide new insights into molecular mechanisms of the disorders related to αSyn.
